# Lived experiences of breast cancer survivors after diagnosis, treatment and beyond: qualitative study

**DOI:** 10.1111/hex.12372

**Published:** 2015-05-07

**Authors:** Faustine Williams, Stephen C. Jeanetta

**Affiliations:** ^1^ Division of Public Health SciencesDepartment of SurgeryWashington University in St. Louis School of MedicineSt. LouisMOUSA; ^2^ Department of Rural SociologyCommunity Development ProgramUniversity of Missouri ExtensionUniversity of MissouriColumbiaMOUSA

**Keywords:** breast cancer, qualitative, survivorship, women

## Abstract

**Background:**

The number of breast cancer survivors has increased since 1990 due to advances in biomedical technology that lead to an increase in early diagnosis and treatment. Research on survivorship has focused on the psychological and treatment aspects of the disease. The goal of this study was focused on exploring the lived experiences of breast cancer survivors from diagnosis, treatment and beyond.

**Objective:**

To understand the lived experiences of women who are breast cancer survivors.

**Design, Setting and Participants:**

A purposive sampling strategy was used to recruit participants from two Missouri cancer centres. A total of 15 women breast cancer survivors were interviewed.

**Findings:**

Three major themes emerged that described the lived experiences of the women. These were factors from the diagnosis and treatment management impacting survivorship, relationship and support system and implication of survivorship. Participants noted that coping with the diagnosis and treatment was a stressful journey and required lots of adjustment and changes. Some developed various techniques such as journaling their activities which provided comfort. In addition, support from family was shared as the key which gave them strength and courage through the different stages of treatment. However, they found it difficult to articulate what survivorship meant.

**Conclusion:**

Using in‐depth interview techniques, this study shed light on the experiences of women who were diagnosed with breast cancer and have completed treatment. They acknowledged frustration with their diagnosis and body changes. Support received from family and friends helped them cope through their treatment. However, they felt abandoned once the treatment phase was over and were uncertain what survivorhood meant to them.

## Background

Breast cancer is the most frequent malignancy affecting women across all ethnic groups in the United States. In 2013, an estimated 232 340 new cases of invasive breast cancer were expected to be diagnosed among women.^1^ At the same time, the number of breast cancer survivors is increasing due primarily to advances in biomedical technology leading to an increase in early diagnosis and treatment. Mortality rates also have been decreasing since 1990, and there are over 2.9 million women in the United States who have survived breast cancer.^2^

Despite the high number of breast cancer survivors, most of the qualitative research on survivorship has focused on the psychological and treatment aspect of the disease.^3^, ^4^, ^5^, ^6^, ^7^, ^8^, ^9^, ^10^, ^11^, ^12^, ^13^ Doumit *et al*.^3^ used a phenomenological approach to study the coping strategies of breast cancer survivors in Lebanon. Participants described their journey with the disease process as a non‐stop fight against cancer. They described cancer as a ‘cut in their lives' that they had to deal with. In spite of the differences in the stage at diagnosis, most participants spoke about similar facilitating and hindering factors for coping. Fu *et al*.^6^ explored and described breast cancer survivors’ experiences of lymphedema‐related symptoms. The women portrayed their experiences as ‘perpetual discomfort’ and ‘frustration’ with their daily lives. There is very limited research on understanding the experiences of breast cancer survivors from the time they were diagnosed with cancer through the process of treatment and life beyond as cancer survivors. The term survivor is defined as life without cancer after treatment. Thus, the purpose of this phenomenological study was to understand the lived experiences of breast cancer ‘survivors’. The central research question was ‘What is it like to be a breast cancer survivor?’ Subquestions were as follows:

1. How do stages of breast cancer diagnosis affect treatment options?
2. What adjustments do breast cancer survivals have to make in their daily life?
3. How do breast cancer survivors perceive quality of life after diagnosis and treatment?

## Method

### Ethics approval

The University of Missouri Institutional Review Board (#1161517) approved the study. Each participant was informed about rights and principles of protection of human subjects, and each approved their participation with a written informed consent under the principle of full disclosure and received a copy of the consent form.

### Selection and recruitment of participants

All the women were recruited from two Missouri cancer centres through the ‘Look Good Feel Better’ (LGFB) group which is a free programme sponsored by the American Cancer Society that teaches beauty (feeling good and fabulous about one's appearance and self‐image during chemotherapy and radiation treatments) techniques to female patients with cancer in active treatment to help them combat the appearance‐related side‐effects of cancer treatment. The programme is open to all women with cancer and undergoing treatment throughout the United States.

A purposive sampling strategy was used by researchers to recruit the participants (*N* = 15). The desire was to have a sample that represented a variety of perspectives but shared some common experiences. The link to the LGFB was the shared experience. Some diversity was sacrificed, but it was decided that a common connection, such as LGFB, was a way to explore how that commonality affected their lived experience. The aim of the purposive strategy was to recruit participants that represented a range of perspectives within the LGFB. A diversity of experiences was based on age at diagnosis, stage at diagnosis and length of survival at time of the interviews. The goal was to get a broad cross section of perspectives within this framework. There were two challenges to this sample frame. One, there were no participants we could identify who would participate in the study who were of low income and without insurance. At fifteen participants, it was clear that we had reached a level of saturation with the population of breast cancer survivors from our potential sample frame. We were no longer getting new information that contributed to our analysis of the breast cancer survivor experience. We could have continued interviewing, but it was clear that any new data were not likely to impact the key themes that had already emerged from the fifteen interviews. The inclusion criteria were women: (i) older than 18 years of age; (ii) English‐speaking; (iii) a diagnosis of breast cancer; and (iv) completion of treatment, surgery, chemotherapy and/or radiation.

### Data collection

Semi‐structured, one‐on‐one in‐depth interviews were conducted with all participants at a location that was most convenient for each. A follow‐up interview was conducted with some individuals to gain further understanding of their experiences and to seek clarification on diagnosis, treatment and life after cancer. Topic guides were developed based on the gaps identified in the cancer literature and the researchers’ experiences working with cancer patients (Table 1). As interviews progressed, participant statements provoked further questions and clarifications. Documents in the form of pictures, memos and posters were also collected from participants and served to help inform the analysis.

**Table 1:** Interview guide

Opening questions
Describe your experience with breast cancer
Tell me about the treatment options that were available to you and why you made that decision
Tell me about the life changes you have made after treatment
Follow‐up questions
After the participant exhausts her response to these opening questions, she will be prompted by the following questions:
Can you describe some of the thoughts or feelings you experienced when you were told you had breast cancer? Who was the first person you told about your diagnosis? What stage was the cancer diagnosed? How was your breast cancer treated? Tell me about the information/advice you were given on what to expert.
What has been the impact of breast cancer on your personal life (e.g. family, friends, socially)?
Recovery from breast cancer takes time. Tell me about activities that helped you during your diagnosis and treatment.
Tell me about life changes you have to make now as a survivor (e.g. home, work, social life).
How has breast cancer changed your overall life?
What are the major challenges you have faced during diagnosis, treatment and now as a survival? Which of these were your biggest challenge? What does being a cancer survival means to you? What kind of support group or network do you belong to? What surprised you about breast cancer What/how will you define survival? And why?
Based on your experience, what do you think would help someone who is just finishing treatment to begin the recovery process? What advice do you have for other women about breast cancer screening? What advice do you have for other women with breast cancer?

### Analysis

The focus of the research was to develop a set of plausible and coherent understandings based on the perspectives of women with breast cancer. Data were analysed following phenomenological coding guidelines.^14^ Analysis of data began after all tapes were transcribed verbatim and reviewed by the research team several times to get familiar with data and expressions of participants in regard to their breast cancer experience. The first level was a line‐by‐line analysis of data (transcriptions from the interview, field notes and document collected). Identified statements were conducted and labelled/highlighted using NVivo 9. Next the invariant constituents were identified by reducing and eliminating overlapping significant statements. From this list, the researchers created meanings bearing in mind not to disrupt the original meaning of the participants. All meaningful units were assigned codes, which were organized into categories of similar codes. The themes were developed by looking across the categories of codes to identify those commonalities that cut across several categories and began to help us better understand the survivor experience.

### Reliability and validity of the study

The following measures were taken by the researchers to ensure the reliability and validity of the instrument and study: (i) the first author who interviewed all participants transcribed tapes verbatim and the first level of analysis, (ii) the second author reviewed the codes and themes to ensure results were true representative of participants’ story/lived experiences, and (iii) authors met to explore differences and refine codes.

## Findings

Ten white and five black breast cancer survivors participated in interviews that lasted from 53 to 75 min. The age of participants ranged from 47 to 69 years. Of the fifteen women, three were diagnosed at stage 0 of the disease, one at stage I, eight at stage II and three women had stage III cancer. Seven women had been cancer free between 2 and 5 years, while eight reported between 6 and 19 years cancer free. All participants had at least a bachelor degree and a full‐time job (Table 2).

**Table 2:** Background information of participants

Participants	Date of diagnosis	Age at diagnosis	Family history of cancer	Stage at diagnosis	Level of education	Treatment finance	Type of treatment	Length of survival (years)
Doreen	2007	48	No	0	College	Insurance	Surgery and radiation	5
Eve	2006	59	No	I	College	Insurance	Double mastectomy	6
Jones	2006	36	Yes^a^	0	College	Insurance	Mastectomy	6
Jenny	2006	56	No	0	College	Insurance	Surgery, chemo and radiation	5
Keila	2008	48	Yes^b^	III	College	Insurance	Double mastectomy	4
MaryAnn	2010	52	No^c^	III	Postgraduate school	Insurance	Double mastectomy	2
Matter	2009	43	Yes^b^	II	Graduate school	Insurance	Double mastectomy	3
Pea	2006	62	No	II	Graduate school	Insurance	Double mastectomy	5
Tracy	2006	61	No	II	Postgraduate school	Insurance	Double mastectomy	6
Survivor 17	1993	42	No	II	Postgraduate school	Insurance	Modified radical mastectomy	19
Piper	2005	35	No	II	College	Insurance	Double mastectomy	7
Rose	2004	40	No	III	College	Insurance	Double mastectomy	8
Pat	2000	34	Yes^b^	II	College	Insurance	Double mastectomy	12
Vivi	2010	40	No	II	Graduate school	Insurance	Double mastectomy	2
Ashley	2005	42	No	II	College	Insurance	Double mastectomy	7

a Family History and Husband.

b Family History.

c Husband, but no Family History.

Average age at diagnosis = 46.5.

Average survival length = 6.5.

Analysis of the interviews identified three major themes that were important to the lived experiences of the women. These were (i) factors from the diagnosis and treatment management impacting survivorship, (ii) relationship and support system and (iii) implication of survivorship.

### Factors from the diagnosis and treatment management impacting survivorship

According to the women, their prior knowledge and understanding of breast health helped them identify changes in their breast and to seek early medical treatment. Pea noted that her annual mammogram examination in July 2006 got rescheduled to October due to scheduling conflict and was about to be rescheduled again. However, she sensed something was not right with her breast.

I just told them you know, I got a lump and it's getting bigger. Well that got them [because at that time], the lump I could feel was the size of walnut. (Pea)

The knowledge and understanding the women had about breast health and cancer became a source of hope and a type of empowerment for the study participants during the diagnosis and treatment management stages of their diagnoses. Doreen discussed the importance of the breast screening and annual mammogram in relation to the new United States Preventive Services Task Force (USPSTF) guideline, which recommended that women have an annual mammogram beginning at age 50 instead of 40, the previous guideline. Doreen believed that the younger age guideline of 40 helped save her life so she was highly critical of the change in the guideline to 50 recommended by USPSTF.

Well, I was 48 so I'm glad it [the new recommendation to wait until 50 to have a mammogram] wasn't in effect when I went through because it wouldn't have been detected…it wouldn't have been stage 0. (Doreen)

Stage at diagnosis and treatment options was another factor which impacted the women's recovery process. Participants indicated they were ‘shocked’ and ‘scared’ when they were informed about the diagnosis.

I was scared because you don't know what to expect, but you keep going… (Piper)

Stage at cancer diagnosis has an impact on an individual's prognosis and survivorship. Participants noted, once the diagnosis was certain they were grateful it was not an aggressive form of breast cancer.

I was just thankful that my cancer was so early, was so treatable. (Jenny)

While most participants shared that their cancer was not detected late, due to fear of recurrence, they chose the most ‘aggressive treatment’.

I was ready for the option of mastectomy and my doctor said, you don't need to be that radical, but it's your choice… I opted for mastectomy. (Tracy)

The body changes experienced by the women were a challenge for them during treatment that several of the participants discussed. They stated that the hair loss was the aspect of treatment that disturbed them the most. It was highly detrimental to their ability to cope and negatively affected their self‐esteem. However, they tried to make the best of the situation and their efforts were illustrated by their resilience during the process. The women indicated it was not funny going through all these experiences, but they tried to find *‘humour’* in the process.

Cost of cancer treatment. All the women in this study had health insurance and were able to access medical care. Participants considered themselves lucky because they did not experience too many financial difficulties because their health insurance covered treatment cost.

…I've heard of people who lost their business and so they are home for family member who had extensive cancer treatment. I didn't have any problem. It costs me very little. (Doreen)

All the women noted they could not have maintained the same quality of life, if they have not had full‐time job and health insurance that paid for the treatment. Nevertheless, they expressed concern for other women who faced the same problem without a job (with insurance) and money to undergo treatment.

My life would have been very terrible. I think it makes it harder to make the same decision. (Ashley)

I will tell you though, if I didn't have insurance, I probably would not have a home right now. I mean, we couldn't have made the payments. (Rose)

According to participants, coping with the diagnosis and treatment of breast cancer was ‘a very stressful journey’ for cancer patients and survivors; this is complicated by ‘fear, anxiety, grief and many emotional issues’. Coping in this context referred to their ability to successfully manage the stress and challenges associated with the diagnosis and treatment of breast cancer. To cope with their diagnosis and treatment required lots of adjustment and changes in order to effectively deal with physical symptoms and treatment impacts. Participants developed various methods that helped them keep their mind off their disease. Some mentioned that journaling their daily activities provided them comfort, while others were involved in various fun activities that helped them maintain positive attitudes.

### Relationship and support system

The women indicated that support was a critical ingredient to their physical and mental health when they faced cancer care. Most essential was the support of family. Friends and co‐workers were also mentioned as very important as one went through the process. Some of these forms of support were nearly as important as family to several of the participants. These support systems provided the women with strength, courage and motivation to successfully manage and navigate the different stages of treatment and adjustments even after they were told they were cancer free. Family and friends support systems were reported by participants to be the most beneficial. A strong family support was the ‘key’ according to participants.

…my husband was very supportive the whole time. (Piper)

My husband was very supportive…he just treated it like I had cold. I am fortunate to have my family support…it's your immediate family that help you through the physical, through your condition, and also, believe it or not through your mental condition. (Pea)

The incredible support that I had from my husband and my boys… makes the treatment itself bearable. (Keila)

As important as support system is, participants mentioned that not all support systems were helpful to them. For instance, Jenny mentioned she advised and encouraged her cousin to join a formal breast cancer support group when she [her cousin] was diagnosed with cancer, but her cousin would not participate. This frustrated Jenny because she could not understand why her cousin would not participate. But after Jenny was diagnosed with cancer, she realized formal support groups may not be as important as they were portrayed in the cancer social support literature. Several of the women shared a similar perspective. They mentioned formal breast cancer support groups were not required for ‘everybody but [worked for] a lot of people’. Several participants seemed to think that formal breast cancer support groups were important but not necessarily a good match for them. They preferred the support they got from family and friends. Some viewed these meetings as depressing.

It was difficult to see younger individuals like 35 or 40 years who don't have a good prognosis like the older ones and have small kids to take care of. (Rose)

…those who have double mastectomy and numerous cancers and are aware they are gonna die; it was sad. (Vivi)

Participants reported it was important for them to have friends going through the same situation at the same time.

…not just friends, but friends going through the same thing. (Pea)

One woman shared that she and another participant became friends during their treatment process. They considered this to be like a sisterhood, creating a bond that would last for the rest of their lives. To them, it was valuable to have had this type of friendship rather than being part of a formal social support group. They shared that they did not need support groups because they went through the stages of the process together. When they were going through the stage where their hair was falling out, they had a hair shaving party together. They did things together throughout the entire process to provide mutual support. Other things included calling each other after chemotherapy and comparing notes.

I had a girlfriend that was diagnosed two weeks before me and another girlfriend diagnosed two weeks after me. So it was the three of us. So if you got to feeling really down, you could call one of them… (Tracy)

### Implication of survivorship

Survivorship is a unique journey and was viewed from a different perspective from survivor to survivor. Diagnosis with breast cancer caused the women in this study to appreciate and realize how ‘precious life is’ and the importance of living life one step at a time and to never to take each passing day for granted. Although the women fully accepted what they went through and were ready to share their experiences and insights on breast cancer, some of them were still not sure what survival meant, and felt they were not ‘quite there yet’.

I just hate the term warriors and the battle cry. Because it's like; what are you fighting? That's exhausting! (Eve)

I don't mind talking about it, but I don't know what to call myself. (Vivi)

On the other hand, some of the women had fully embraced themselves as ‘warriors’ and are ‘grateful to God to be alive’. MaryAnn noted that to her, ‘Survivor means a day without cancer’.

Others shared that to them survivorship meant development of new attitudes and perceptions about life in general.

I will say it helped to [become] more centered. That I'm more in tune with myself and what I want to do and what I'm capable of doing. (Doreen)

Another aspect of the survivorship journey participants shared focused on advocacy and foundation creation. Participants indicated that breast cancer is a disease that all women could identify with irrespective of age, colour and education. Many women in this study were involved with foundations and other projects to provide education, generate awareness as well as raise funds for breast cancer research. A few of the women who were participants of this study shared a desire to establish a foundation of their own but most did not. One participant, Doreen, expressed an interest in raising funds but was not interested in any activism beyond that:

…I think that might be the extent of my helping raise funds right now. (Doreen)

A majority of the other participants were not all enthusiastic about creating foundations in their names. They believed in the principle of doing things in the ‘quiet’ rather than getting involved in activities for ‘splashy attention’. They shared that the creation of a foundation was not for them.

I don't like to be in the lime‐light or anything like that. I will give money to a foundation towards breast cancer survivors. (Pat)

…I don't like to be reminded I've had breast cancer… (Ashley)

## Discussion

The purpose of the study was to better understand what it meant to be a cancer survivor by exploring the lived experiences of women who were ‘cancer survivors’ as they moved through the diagnosis, treatment and recovery process. Several things emerged from this analysis that can provide insight as to what it meant to be a cancer survivor and how different elements of the treatment process affected their perceptions of the survival experience.

Individuals who have lived past cancer are often referred to as ‘survivors’. Does every breast cancer women want to be called a survivor? Previous studies on survivorship reported that women embraced their new identity.^15^, ^16^, ^17^, ^18^, ^19^, ^20^, ^21^ Writing on the ideological dimension of cancer Perusek^22^ challenged the language of ‘survivorhood’. He reported that while cancer survivors existed, he believed the word did injustice to his actual experience as a cancer‐free person. However, according to Frank^23^, the designation of ‘survivor’ did not encompass the rich experiences of cancer‐free patients. As a cancer‐free person, he noted that initially he felt ‘survivorhood’ was untenable, but later made peace with himself and later accepted the designation because it was ‘inescapably widespread’. Moreover it ‘fit’ so well even though he felt sometimes the label was too bare to describe the rich experiences of survivors.^23^

In the present study, apart from one woman who embraced her new identity as a ‘warrior and survivor’, the rest of the women in this study did not see themselves as warriors/survivors, but were thankful to God they were cancer free. They found it difficult to articulate what survivorship meant. The women actually found the transition from being a ‘cancer patient’ to being a ‘cancer survivor’ very abrupt. One day, they were told they did not have to come back/as often, which resulted in a rather dramatic decline in their perception of the kind of support that would be available going forward. While the medical treatment may have been finished, the recovery process was obviously not complete for these women. The support they received from the health‐care providers changed, and in some cases, the expectations of family and friends also changed. They articulated a need for some support beyond post‐treatment to help deal with on‐going fear, and the transition to a more normal life. This finding was consistent with Frank^23^ report which stated that cancer survivors felt abandoned by their families as soon as treatment was over. Given that support systems were critical to coping with the diagnosis and treatment, on‐going support, particularly from family and friends, would be critical to successfully transitioning to life after cancer.

It was clear that managing fear was an important part of the diagnosis, treatment and recovery process. The breast cancer experience elicited many fears and concerns among the women interviewed as they progressed through the process. These included fears and concerns related to potential disfigurement from surgery, loss of body image, loss of sexuality and life after treatment, especially the possible recurrence of cancer. Learning how to manage the fear of recurrence was also part of what it meant to be a survivor. Some of the women chose to have more invasive surgeries because they were afraid of a potential recurrence of the cancer even when the medical staff assured them the probability was very low. They chose this option because they wanted to eliminate the risk to the greatest extent possible. They did not want to have to face the cancer again and chose the most extreme treatment possible even when the risk was low. This was also an option for them given they had the insurance and resources to make this choice.

The women in this study expressed how important information and knowledge of breast cancer risks was to their decision‐making processes as they progressed through the treatment and recovery process. It helped them feel empowered to make the best decisions they could concerning their health. The women in this study stressed that women should never take their annual mammogram examination for granted. Several of the women fervently believed that their survival was enhanced because of their vigilance in terms of keeping up regular examinations. They also noted that when a woman chose to attend mammography, she voluntarily agreed to undergo breast screening. However, this did not imply that she has knowledge about her body. Participants mentioned it was required of all women to be familiar with their body in order to note changes and seek early medical attention. They found body knowledge empowering and several were diagnosed early because of the knowledge they had of their bodies. The process of becoming a cancer survivor reinforced this value and was a legacy of their survivorship.

Consistent with the literature, social support was critical during the progress of diagnosis, treatment and beyond for the women.^3^, ^13^, ^15^, ^16^, ^17^, ^24^ Support from family, peers, co‐workers and especially cosurvivors and/or cocancer patients were reported to be most beneficial. Participants noted that the positive support received from these groups of individuals not only helped them accept their condition but also gave them the hope and encouragement they needed to engage in their fight against cancer.

In contrast with previous findings on formal cancer support groups,^3^, ^13^, ^15^, ^16^ the women in this study did not find them useful. This finding may be unique to this study, but some of the factors they found challenging about the process would likely be issues for others too and warrant further investigation. For example, the women said such meetings were perceived as depressing, boring and lifeless. Some suggested that because they had a better prognosis than others in the group, they actually felt guilty for being in a better situation. This finding suggests clinicians should look more closely at a range of support options for women that included support groups but may include access to alternatives such as peer mentors and friend networks that were more intimate and linked to people in similar circumstances and stages of treatment.

Stage of diagnosis and treatment was another important theme that emerged. This was consistent with the literature which stated, cancer treatment and survival depended largely on the stage and prognosis of the disease.^3^, ^15^, ^16^, ^25^ The women in this study were acutely aware that their survival was enhanced because of early detection. The earlier the cancer was detected, the better quality of health and the greater the number of years they could expect to survive. Participants in this study were surprised by the USPSTF guideline that women in their 40s should stop routinely having annual mammogram.^26^ Some took it personally as they felt their own survival was greatly enhanced because of early detection. As survivors, they had friends, family members and work colleagues who they did not want to see go through the same experience and expressed concerns about what the changes would have meant for them and their survivorship had these changes been in effect when they were diagnosed with cancer. It tended to threaten their sense of security and ability to take action on their own behalf. For some of the women in this study, the new guidelines were a step backwards and served as a real threat to early detection. Participants in this study encouraged all women in their 40s not to ignore their annual mammogram examination.

The physical change in appearance, specifically the hair loss (eyelashes and eyebrow), was a major negative factor affecting the ability of these women to effectively cope in the initial stages of their diagnoses and treatment. This was similar to what was reported by Doumit *et al*.,^3^ Beatty *et al*.^11^ and Pelusi *et al*.^27^ Some described it as loss of control, but the knowledge and understanding that they were lucky, and it could have been worse, helped the women turn their frustrations into humour as they tried to have fun with it. When they reflected on the experience as survivors, it was the stories they told of their coping strategies that helped shape their own role in the process. It was something they could control during the treatment process. As survivors, the stories of their own coping was what made the experience of going through the treatment process easier to discuss.

Cancer patients whether insured or not face significant and sometimes devastating hurdles in regard to receiving timely and affordable treatment.^28^ Women in this study did not experience any financial difficulties with their own cancer treatments, which is not true of many patients with breast cancer. Participants expressed fear and concern for those who are poor and have no insurance coverage. They were concerned these women will be unable to obtain the needed treatment and care.^16^ Participants noted, they could not have maintained the same quality of life, without the health insurance that paid for their treatment.

### Clinical implications of the study

Much of the breast cancer research has focused on the physical aspects from medical perspective without addressing the stories of the survivors’ lived experiences. Using in‐depth interviews, and listening to women, we have shared their experiences with diagnosis, treatment and life after cancer. This study therefore serves as a tool to voice the lived experiences of these women by enhancing our understanding of the challenges these women faced as they completed the treatment process and continued their lives as ‘survivors’. It was clear from the stories they shared that survivorship included learning how to live with the experience and manage the fear of recurrence, which according to these women never goes away. Another implication of this study relates to education and counselling. Most of the women interviewed in this study did not want to be called breast cancer ‘survivors’ or ‘warriors’ which is a common term for someone who has been diagnosed and treated for breast cancer. For breast cancer educators and counsellors, this study hopes to further their knowledge and understanding regarding the use of the word survivor when counselling women who have experienced the disease or have completed treatment and were cancer free. The transition from cancer patient to cancer survivor has not been viewed as a process. Clinically, when the cancer is gone, then the patient is finished. However, the women expressed a sense of abandonment as a result. Additional work needs to be done to better understand how to effectively help women transition from a treatment phase into a recovery phase that helps them better manage life after cancer because the process haunted many of these women long after the cancer was gone.

### Limitations of the study

The main limitation of this study is that the experiences associated with these women may not reflect the experiences of all breast cancer survivors. All participants in the study are well‐educated, have stable families, economic resources and health insurance and did not experience much financial difficulties regarding cost of treatment that many women face which points to the need to broaden the scope of this work. One of the challenges as a researcher is to reach the women who are difficult to access. For those without insurance, their lack of resources and marginalization in the health‐care system made it much more difficult to engage them in this type of research process.

## Conclusion

According to Frank^29^, narrative stories on diseases can complement medicine by teaching others how to live and cope with illness. Using in‐depth interviews and analysing the experiences of these fifteen women, the authors shared the experiences of these women with diagnosis, treatment and beyond. Three main themes were identified as a way to begin to address an area that has not been adequately studied and should be further explored as it relates to the diagnosis treatment and survival of breast cancer. This study shed some light on the experiences of the women who completed cancer treatment and were recovering from the process. This study hopes to provide some voice to the experiences of these women and inform health‐care providers about the challenges these women faced. The women acknowledged to have experienced frustration with their diagnosis and body changes. However, the shared support received from family and friends helped them cope through their treatment. At the same time, they felt abandoned once the treatment phase was over and were still uncertain what survivorhood meant to them.

## Source of funding

None.

## Conflicts of interest

None declared.
